# RETRACTION: Fermented Mistletoe Extract as a Multimodal Antitumoral Agent in Gliomas

**DOI:** 10.1155/ecam/9764712

**Published:** 2026-02-24

**Authors:** Evidence-Based Complementary and Alternative Medicine

RETRACTION: O. Podlech, P. N. Harter, M. Mittelbronn, S. Pöschel, and U. Naumann, “Fermented Mistletoe Extract as a Multimodal Antitumoral Agent in Gliomas,” *Evidence-Based Complementary and Alternative Medicine* 2012, no. 1 (2012): 501796, https://doi.org/10.1155/2012/501796.

The above article, published online on 22 October 2012 in Wiley Online Library (https://wileyonlinelibrary.com), has been retracted by John Wiley & Sons Ltd. The retraction has been agreed after unexpected similarities were noted between the panels, ‘ISCADOR M ‐ 24 hr’ and ‘ISCADOR Q ‐ 24 hrs’, in Figure 5b. In addition, unexpected similarities were noted between the blots, ‘MMP‐1 ‐ Ponc.’ and ‘TIMP‐2 ‐ Ponc.’, in Figure 6a.

Several attempts by the journal to contact the authors did not result in a reply. As such, the results and conclusions for this article cannot be considered reliable.

